# Seismic response analysis of subway station under obliquely incident SV waves

**DOI:** 10.1038/s41598-024-59593-4

**Published:** 2024-04-21

**Authors:** Hui Zhu, Songhong Yan, Weiyu Sun, Rongling Zhang, Erfeng Ou, Qingguo Liang

**Affiliations:** 1 School of Civil Engineering, Lanzhou Jiaotong University, Lanzhou, 730070 China; 2 Key Laboratory of Road & Bridge and Underground Engineering of Gansu Province, Lanzhou, 730070 China; 3 National and Local Joint Engineering Laboratory for Disaster Prevention and Control Technology of Road and Bridge Engineering, Lanzhou, 730070 China

**Keywords:** Engineering, Civil engineering

## Abstract

This paper aims to investigate the dynamic response characteristics of subway station under earthquakes. To this end, seismic waves are transformed into equivalent nodal loads on viscoelastic artificial boundaries using theories and methods of wave motion. The calculation formulas for equivalent nodal loads of SV waves incident at any angle are established, and ANSYS' APDL program compiles to automatically generate the viscoelastic artificial boundary and input the seismic loads. A finite element model of soil-subway station interaction was established, and the seismic response characteristics of a two-story three-span subway station under different incidence angles of SV waves were investigated using the above seismic input method. The results indicate that the incidence angle of seismic waves has a significant impact on the seismic response of subway station. Inclined incidence of seismic waves causes non-uniform loading and deformation of the subway station. Specifically, a small angle leads to predominantly transverse shear deformation, while a large angle causes mainly vertical shear deformation. The inclined incidence of seismic waves significantly increases the vertical acceleration of the subway station, with the effect becoming more pronounced as the angle increases. Additionally, special attention should be given to the joints between the structural slab and the side wall, slab and center column, as well as the two ends of the center column as they are vulnerable areas during earthquakes and require careful consideration in seismic design.

## Introduction

The seismic performance of underground structures is usually deemed superior to that of surface structures because of the constraints imposed by the surrounding rock and soil bodies. That being said, various inquiries of strong earthquakes in recent times have evidenced the serious harm that such tremors can inflict upon underground structures, thus imperilling the safety of both city traffic operations and individuals' lives and properties. For instance, many subway stations and tunnels in Kobe City suffered significant damage during the Hanshin earthquake of 7.2 magnitude in Japan in 1995^1–5^. Similarly, around a hundred tunnels sustained serious damage during the Wenchuan earthquake of magnitude 8.0 in China in 2008^6–10^. More recently, in Qinghai Province, China in 2022, the Menyuan earthquake severely damaged the Daliang Tunnel^11,12^, while the Turkey earthquake of magnitude 7.8 in 2023 caused harm to the Erkenek tunnel^13^. Numerous instances of earthquake damage have resulted in substantial economic losses. Furthermore, they serve as a warning for the need to pay more attention to the seismic problems of underground structures.

Currently, significant progress has been made in seismic research of subway station structures^14–18^. Regarding numerical simulations, Du et al.^19^ constructed a three-dimensional finite element model of Dakai subway station and investigated its failure mechanism under horizontal earthquake action alone as well as combined horizontal and vertical earthquake action. The researchers concluded that the loss of shear resistance in the shallow underground structure's overburden, due to strong earthquake action, would result in the middle column experiencing both vertical inertial force and horizontal shear load. Eventually, the compression shear failure of the middle column leads to the collapse of the structure. Zhuang and colleagues^20^ utilized a two-dimensional finite element model to perform numerical calculations and analyze the nonlinear seismic response of Dakai subway station. They also presented the damage evolution process of the station during the Hanshin earthquake. The numerical analysis results were consistent with the actual earthquake damage and effectively elucidated the observed damage phenomena at Dakai subway station. Tian^21^ conducted seismic research on a station structure along Beijing Metro Line 7, using both spectral analysis and dynamic time history calculations. She found that during horizontal earthquake activity, the main bearing member of the subway station structure is the middle column, with large axial force, and the roof structure is prone to failure due to large deformation and stress. In terms of model testing, Chen et al.^22^ conducted a large-scale shaking table experiment to analyze the dynamic response of subway station structures in deep soft soil fields. Their results revealed that the middle column exhibited greater strain response compared to other components, while the top of the side wall experienced more strain than the bottom. Li and colleagues^23^ conducted a shaking table experiment to investigate the impact of bi-directional earthquake action on the structure of pillar-free subway stations. This study addressed a gap in domestic research on the topic. Ling et al.^24^, through centrifugal shaking table experiments, simulated the failure process of subway station structures under earthquake conditions. The findings indicate that during the incident, the columns within the station structure face increased axial pressure due to the overlying soil and self-weight, thereby emerging as the vulnerable component of the station structure prone to failure. It is observed that the aforementioned studies assume that seismic waves have only perpendicular or horizontal incidence, without taking into account the case of inclined incidence. This hypothesis holds true for deep earthquakes or soil and other similar mediums. Yet, for shallow earthquakes, or rock masses, the angle of seismic waves changes gradually during propagation due to the close proximity of the source and the medium's high stiffness. Thus, for underground structures, seismic waves tend to incident at a certain angle, and the resulting non-uniform deformation has a significant impact on the laws governing the seismic response.

Therefore, this research paper utilizes the concept of the viscoelastic artificial boundary to convert the seismic wave into an equivalent load applied to the artificial boundary node. To enable the incidence of SV waves at any desired angle, the APDL program is developed and integrated into the powerful general finite element software, ANSYS. Consequently, a shallow buried two-story three-span subway station structure is selected as a representative example to examine the seismic response characteristics under various incident angles of SV waves.

## Oblique incident seismic wave input method

### Viscoelastic artificial boundary

**Figure 1:**
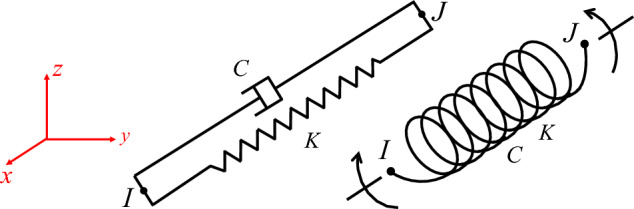
Geometry of the COMBIN14 element.

**Figure 2:**
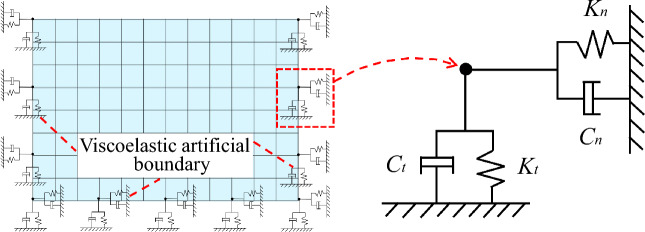
Viscoelastic artificial boundary diagram.

When establishing the finite element model, it is crucial to select an artificial boundary that can accurately simulate infinite ground characteristics and correspond with ground motion input. This selection is pivotal in representing the overall dynamic characteristics of soil-structure with precision. The viscoelastic artificial boundary overcomes the limitations of low frequency drift of the viscous boundary and high frequency instability of the transmitting boundary, exhibiting excellent stability at both low and high frequencies. Additionally, it can simulate the elastic recovery performance of a semi-infinite foundation, making it a frequently used artificial boundary by numerous scholars^25–32^. The COMBIN14 element, which includes a spring element and a damper element, can be utilized within the ANSYS finite element software to model the viscoelastic artificial boundary. Figure 1 displays the element's geometry^33^ while Fig. 2 depicts the artificial boundary's schematic diagram.

The relevant parameters of the spring-damper system in viscoelastic artificial boundary are:

1 $$ \left\{ \begin{gathered} K_{n} = \alpha_{n} G/R \hfill \\ C_{n} = \rho C_{p} \hfill \\ \end{gathered} \right. $$

2 $$ \left\{ \begin{gathered} K_{t} = \alpha_{t} G/R \hfill \\ C_{t} = \rho C_{s} \hfill \\ \end{gathered} \right. $$

where $K_{n}$ and $K_{t}$ represent the stiffness coefficients for the normal and tangential springs, respectively, $C_{n}$ and $C_{t}$ denote the damping coefficients for the normal and tangential dampers, respectively. *R* represents the distance from the wave source to the artificial boundary node. For regular models, the wave source is typically located at the geometric center of the model, thus *R* can be calculated as $R = \sqrt {L^{2} /4 + H^{2} /4}$, where *L* is the width of the model and *H* is the height of the model. *G* indicates the shear modulus of the infinite domain medium, while *ρ* denotes its density. $\alpha_{n}$ and $\alpha_{t}$ represent the normal and tangential artificial boundary correction coefficients, with values taken from the research of Liu et al^35,36^ as 1.0 and 0.5, respectively. Additionally,$C_{p}$ and $C_{s}$ represent the propagation velocities of P and SV waves, respectively, in the infinite domain medium, which can be calculated according to Eq. (3):

3 $$ \left\{ \begin{gathered} C_{p} = \sqrt {\frac{\lambda + 2G}{\rho }} = \sqrt {\frac{{E\left( {1 - \mu } \right)}}{{\rho \left( {1 + \mu } \right)\left( {1 - 2\mu } \right)}}} \hfill \\ C_{s} = \sqrt {\frac{G}{\rho }} = \sqrt {\frac{E}{{2\rho \left( {1 + \mu } \right)}}} \hfill \\ \end{gathered} \right. $$

### The equivalent node load on the artificial boundary

**Figure 3:**
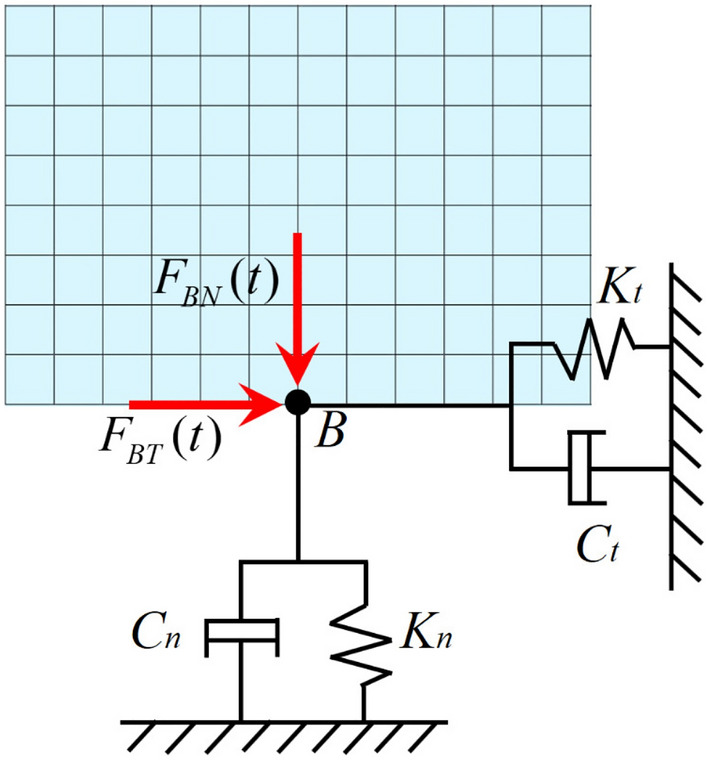
Diagram of viscoelastic artificial boundary equivalent nodal loads.

The total wave field at the viscoelastic artificial boundary is highly complex. By transforming the ground motion input problem into the seismic wave source problem, the total wave field at the artificial boundary can be decomposed into the free wave field (also known as input wave field) and the scattered wave field (referred to as radiation wave field)^34^. The free wave field refers to the known or can be obtained wave field that needs to be input into the finite element model, whereas the scattered wave field refers to the unknown wave field that must be determined via the infinite domain simulation method. Among them, the free wave field needs to be converted into the equivalent node load and inputted into the calculation model, and the equivalent load acting on the artificial boundary node is shown in Fig. 3. While the scattered wave field can be directly absorbed by the viscoelastic artificial boundary, without the need for separate calculation^35,36^.

**Figure 4:**
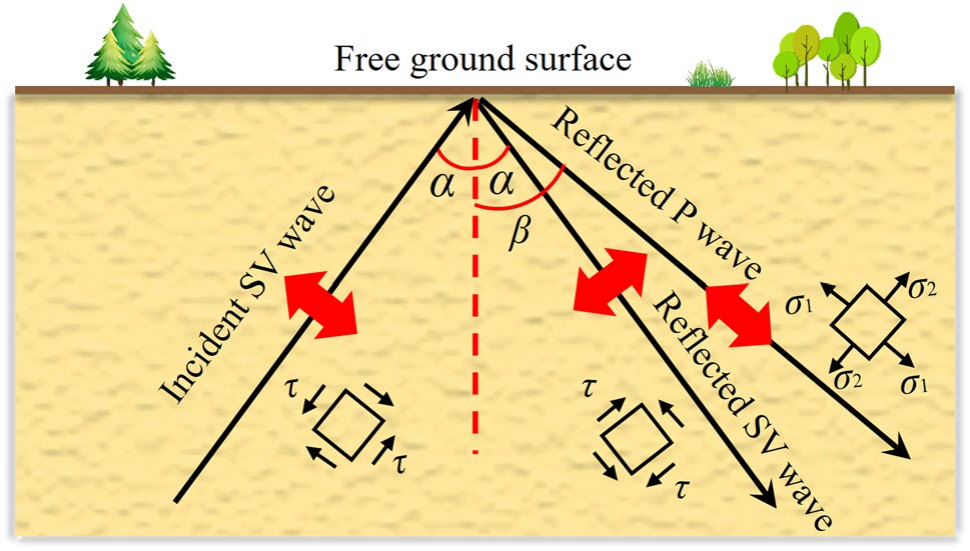
Reflection diagram of SV wave on the free surface.

As shown in Fig. 4, assuming that there is a plane SV wave propagating upward at an angle *α*, it is reflected by the free surface of the ground to produce a plane SV wave with a reflection angle of *α* and a plane P wave with a reflection angle of *β*, where *α* < *β*. The main parameters of wave reflection and refraction are shown in Eqs. (4), (5), (6), (7) and (8).

4 $$ \beta = \arcsin \left( {\frac{{C_{p} \sin \alpha }}{{C_{s} }}} \right) $$

5 $$ k = \frac{\sin \beta }{{\sin \alpha }} = \frac{{C_{p} }}{{C_{s} }} $$

6 $$ B_{1} = \frac{{\sin 2\alpha \sin 2\beta - k^{2} \cos^{2} 2\alpha }}{{\sin 2\alpha \sin 2\beta + k^{2} \cos^{2} 2\alpha }} $$

7 $$ B_{2} = \frac{2k\sin 2\alpha \cos 2\alpha }{{\sin 2\alpha \sin 2\beta + k^{2} \cos^{2} 2\alpha }} $$

8 $$ \alpha \le \alpha_{cr} = \arcsin \left( {\frac{{C_{s} }}{{C_{p} }}} \right) $$

where* β* represents the angle at which the P wave is reflected, *k* stands for the ratio of P wave speed to SV wave speed,$B_{1}$ represents the amplitude ratio of reflected SV wave to incident SV wave,$B_{2}$ represents the amplitude ratio of reflected P wave to incident SV wave; $\alpha_{cr}$ represents the critical value of the incidence angle *α*, where $\beta < 90^{ \circ }$, requiring $\alpha \le \alpha_{cr}$.

**Figure 5:**
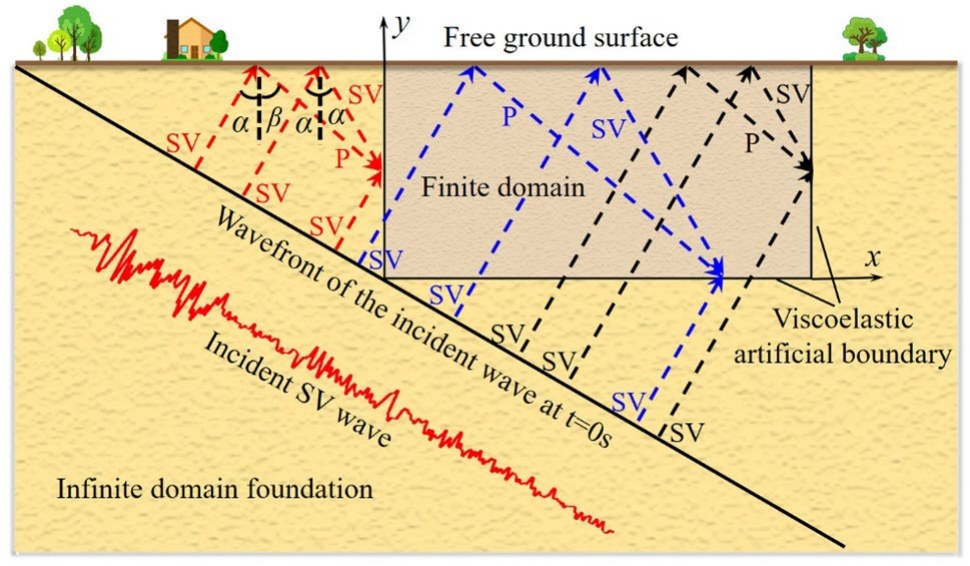
Two-dimensional diagram of plane SV-wave oblique incidence.

Based on the reflection law of SV waves at a free surface, it is possible to obtain the method for decomposing the free wave field on the finite element model when SV waves are obliquely incident, as demonstrated in Fig. 5. The free wave field for any node *b* at the left, right, and bottom boundaries is formed through a superposition of SV wave with an incidence angle *α*, SV wave with a reflection angle *α*, and P wave with a reflection angle *β*.

If node b of the viscoelastic artificial boundary is subjected to an equivalent node load ${\text{F}}_{{\text{b}}}$, Eqs. (9), (10) and (11) can be utilized to calculate the corresponding equivalent node load components of each boundary. Specifically, for the left artificial boundary:

9 $$ \left\{ \begin{gathered} F_{bx}^{ - x} (t) = \left[ {K_{n} u_{bx}^{ - x} (t) + C_{n} \dot{u}_{bx}^{ - x} (t) + \sigma_{x}^{ - x} } \right]A_{b} \hfill \\ F_{by}^{ - x} (t) = \left[ {K_{t} u_{by}^{ - x} (t) + C_{t} \dot{u}_{by}^{ - x} (t) + \tau_{xy}^{ - x} } \right]A_{b} \hfill \\ \end{gathered} \right. $$

for bottom edge artificial border:

10 $$ \left\{ \begin{gathered} F_{bx}^{ - y} (t) = \left[ {K_{t} u_{bx}^{ - y} (t) + C_{t} \dot{u}_{bx}^{ - y} (t) + \tau_{yx}^{ - y} } \right]A_{b} \hfill \\ F_{by}^{ - y} (t) = \left[ {K_{n} u_{by}^{ - y} (t) + C_{n} \dot{u}_{by}^{ - y} (t) + \sigma_{y}^{ - y} } \right]A_{b} \hfill \\ \end{gathered} \right. $$

and for the right artificial boundary:

11 $$ \left\{ \begin{gathered} F_{bx}^{x} (t) = \left[ {K_{n} u_{bx}^{x} (t) + C_{n} \dot{u}_{bx}^{x} (t) + \sigma_{x}^{x} } \right]A_{b} \hfill \\ F_{by}^{x} (t) = \left[ {K_{t} u_{by}^{x} (t) + C_{t} \dot{u}_{by}^{x} (t) + \tau_{xy}^{x} } \right]A_{b} \hfill \\ \end{gathered} \right. $$

where the superscript indicates the direction of the outer normal of the boundary where node *b* is located, while the subscript represents the direction of the load component of node *b*, which is " + " in the same direction as the axis, and "-" in the opposite direction as the axis.$A_{b}$ refers to the area impacted by node *b*, obtainable through the use of the ARNODE function in ANSYS.

According to the given formula, to solve the equivalent node load of node b on a certain boundary, it is only necessary to solve its displacement, velocity and stress field components.

### Solution for displacement, velocity, and stress fields at each boundary

It is assumed that the displacement time history for the oblique incident SV wave is represented by $u_{0} \left( t \right)$ and the velocity time history by $\dot{u}_{0} \left( t \right)$. Due to the delayed arrival of the wave at each boundary during propagation, it is necessary to consider the delay time of each incident and reflected wave arriving at each boundary.

In the case of the left boundary, the free field is composed of the incident SV wave with delay time $\Delta t_{1}$, the reflected SV wave with delay time $\Delta t_{2}$, and the reflected P wave with delay time $\Delta t_{3}$. The displacement field is located is as follows:

12 $$ \left\{ \begin{gathered} u_{bx}^{ - x} (t) = u_{0} \left( {t - \Delta t_{1} } \right)\cos \alpha - B_{1} u_{0} \left( {t - \Delta t_{2} } \right)\cos \alpha + B_{2} u_{0} \left( {t - \Delta t_{3} } \right)\sin \beta \; \hfill \\ u_{by}^{ - x} (t) = - u_{0} \left( {t - \Delta t_{1} } \right)\sin \alpha - B_{1} u_{0} \left( {t - \Delta t_{2} } \right)\sin \alpha - B_{2} u_{0} \left( {t - \Delta t_{3} } \right)\cos \beta \; \hfill \\ \end{gathered} \right. $$

the velocity field is located is as follows:

13 $$ \left\{ \begin{gathered} \dot{u}_{bx}^{ - x} (t) = \dot{u}_{0} \left( {t - \Delta t_{1} } \right)\cos \alpha - B_{1} \dot{u}_{0} \left( {t - \Delta t_{2} } \right)\cos \alpha + B_{2} \dot{u}_{0} \left( {t - \Delta t_{3} } \right)\sin \beta \hfill \\ \dot{u}_{by}^{ - x} (t) = - \dot{u}_{0} \left( {t - \Delta t_{1} } \right)\sin \alpha - B_{1} \dot{u}_{0} \left( {t - \Delta t_{2} } \right)\sin \alpha - B_{2} \dot{u}_{0} \left( {t - \Delta t_{3} } \right)\cos \beta \hfill \\ \end{gathered} \right. $$

the stress field is located is as follows:

14 $$ \left\{ \begin{gathered} \sigma_{x}^{ - x} (t) = \frac{G\sin 2\alpha }{{C_{s} }}\left[ {\dot{u}_{0} \left( {t - \Delta t_{1} } \right) - B_{1} \dot{u}_{0} \left( {t - \Delta t_{2} } \right)} \right] + B_{2} \frac{{\lambda + 2G\sin^{2} \beta }}{{C_{p} }}\dot{u}_{0} \left( {t - \Delta t_{3} } \right) \hfill \\ \tau_{xy}^{ - x} (t) = \frac{G\cos 2\alpha }{{C_{s} }}\left[ {\dot{u}_{0} \left( {t - \Delta t_{1} } \right) + B_{1} \dot{u}_{0} \left( {t - \Delta t_{2} } \right)} \right] - B_{2} \frac{G\sin 2\beta }{{C_{p} }}\dot{u}_{0} \left( {t - \Delta t_{3} } \right) \hfill \\ \end{gathered} \right. $$

and the time solution is located is as follows:

15 $$ \left\{ \begin{gathered} \Delta t_{1} = y_{b} \cos \alpha /C_{s} \hfill \\ \Delta t_{2} = \left( {2H - y_{b} } \right)\cos \alpha /C_{s} \hfill \\ \Delta t_{3} = \left( {H - y_{b} } \right)/\left( {C_{p} \cos \beta } \right) + \;\left[ {H - \left( {H - y_{b} } \right)\left. {\tan \alpha \tan \beta } \right]\cos \alpha /C_{s} } \right. \hfill \\ \end{gathered} \right. $$

where $x_{b}$ and $y_{b}$ represent the x and y coordinates, respectively, of a node b on the boundary; *L* and *H* denote the width and height of the model, respectively. (same below).

In the case of the bottom boundary, the free field is composed of the incident SV wave with delay time $\Delta t_{4}$, the reflected SV wave with delay time $\Delta t_{5}$, and the reflected P wave with delay time $\Delta t_{6}$. The displacement field is located is as follows:

16 $$ \left\{ \begin{gathered} u_{bx}^{ - y} (t) = u_{0} \left( {t - \Delta t_{4} } \right)\cos \alpha - B_{1} u_{0} \left( {t - \Delta t_{5} } \right)\cos \alpha + B_{2} u_{0} \left( {t - \Delta t_{6} } \right)\sin \beta \hfill \\ u_{by}^{ - y} (t) = - u_{0} \left( {t - \Delta t_{4} } \right)\sin \alpha - B_{1} u_{0} \left( {t - \Delta t_{5} } \right)\sin \alpha - B_{2} u_{0} \left( {t - \Delta t_{6} } \right)\cos \beta \hfill \\ \end{gathered} \right. $$

the velocity field is located is as follows:

17 $$ \left\{ \begin{gathered} \dot{u}_{bx}^{ - y} (t) = \dot{u}_{0} \left( {t - \Delta t_{4} } \right)\cos \alpha - B_{1} \dot{u}_{0} \left( {t - \Delta t_{5} } \right)\cos \alpha + B_{2} \dot{u}_{0} \left( {t - \Delta t_{6} } \right)\sin \beta \hfill \\ \dot{u}_{by}^{ - y} (t) = - \dot{u}_{0} \left( {t - \Delta t_{4} } \right)\sin \alpha - B_{1} \dot{u}_{0} \left( {t - \Delta t_{5} } \right)\sin \alpha - B_{2} \dot{u}_{0} \left( {t - \Delta t_{6} } \right)\cos \beta \hfill \\ \end{gathered} \right._{{\phantom{a}}} $$

the stress field is located is as follows:

18 $$ \left\{ \begin{gathered} \tau_{yx}^{ - y} (t) = \frac{G\cos 2\alpha }{{C_{s} }}\left[ {\dot{u}_{0} \left( {t - \Delta t_{4} } \right) + B_{1} \dot{u}_{0} \left( {t - \Delta t_{5} } \right)} \right] - B_{2} \frac{G\sin 2\beta }{{C_{p} }}\dot{u}_{0} \left( {t - \Delta t_{6} } \right) \hfill \\ \sigma_{y}^{ - y} (t) = - \frac{G\sin 2\alpha }{{C_{s} }}\left[ {\dot{u}_{0} \left( {t - \Delta t_{4} } \right) - B_{1} \dot{u}_{0} \left( {t - \Delta t_{5} } \right)} \right] + B_{2} \frac{{\lambda + 2G\cos^{2} \beta }}{{C_{p} }}\dot{u}_{0} \left( {t - \Delta t_{6} } \right) \hfill \\ \end{gathered} \right. $$

and the time solution is located is as follows:

19 $$ \left\{ \begin{gathered} \Delta t_{4} = x_{b} \sin \alpha /C_{s} \hfill \\ \Delta t_{5} = \left( {2H + x_{b} \tan \alpha } \right)\cos \alpha /C_{s} \hfill \\ \Delta t_{6} = H/\left( {C_{p} \cos \beta } \right) + \left[ {H\cos \alpha + x_{b} \sin \alpha - \left. {H\tan \beta \sin \alpha } \right]\;/C_{s} } \right.\;\;\; \hfill \\ \end{gathered} \right. $$

In the case of the right boundary, the free field is composed of the incident SV wave with delay time $\Delta t_{7}$, the reflected SV wave with delay time $\Delta t_{8}$, and the reflected P wave with delay time $\Delta t_{9}$. The displacement field is located is as follows:

20 $$ \left\{ \begin{gathered} u_{bx}^{x} (t) = u_{0} \left( {t - \Delta t_{7} } \right)\cos \alpha - B_{1} u_{0} \left( {t - \Delta t_{8} } \right)\cos \alpha + B_{2} u_{0} \left( {t - \Delta t_{9} } \right){\text{sin}}\beta \hfill \\ u_{by}^{x} (t) = - u_{0} \left( {t - \Delta t_{7} } \right)\sin \alpha - B_{1} u_{0} \left( {t - \Delta t_{8} } \right)\sin \alpha - B_{2} u_{0} \left( {t - \Delta t_{9} } \right)\cos \beta \hfill \\ \end{gathered} \right. $$

the velocity field is located is as follows:

21 $$ \left\{ \begin{gathered} \dot{u}_{bx}^{x} (t) = \dot{u}_{0} \left( {t - \Delta t_{7} } \right)\cos \alpha - B_{1} \dot{u}_{0} \left( {t - \Delta t_{8} } \right)\cos \alpha + B_{2} \dot{u}_{0} \left( {t - \Delta t_{9} } \right){\text{sin}}\beta \hfill \\ \dot{u}_{by}^{x} (t) = - \dot{u}_{0} \left( {t - \Delta t_{7} } \right)\sin \alpha - B_{1} \dot{u}_{0} \left( {t - \Delta t_{8} } \right)\sin \alpha - B_{2} \dot{u}_{0} \left( {t - \Delta t_{9} } \right)\cos \beta \hfill \\ \end{gathered} \right. $$

the stress field is located is as follows:

22 $$ \left\{ \begin{gathered} \sigma_{x}^{x} (t) = - \frac{G\sin 2\alpha }{{C_{s} }}\left[ {\dot{u}_{0} \left( {t - \Delta t_{7} } \right) - B_{1} \dot{u}_{0} \left( {t - \Delta t_{8} } \right)} \right] - B_{2} \frac{{\lambda + 2G\sin^{2} \beta }}{{C_{p} }}\dot{u}_{0} \left( {t - \Delta t_{9} } \right) \hfill \\ \tau_{xy}^{x} (t) = - \frac{G\cos 2\alpha }{{C_{s} }}\left[ {\dot{u}_{0} \left( {t - \Delta t_{7} } \right) + B_{1} \dot{u}_{0} \left( {t - \Delta t_{8} } \right)} \right] + B_{2} \frac{G\sin 2\beta }{{C_{p} }}\dot{u}_{0} \left( {t - \Delta t_{9} } \right) \hfill \\ \end{gathered} \right. $$

and the time solution is located is as follows:

23 $$ \left\{ \begin{gathered} \Delta t_{7} = y_{b} \cos \alpha /C_{s} + L\sin \alpha /C_{s} \hfill \\ \Delta t_{8} = \left( {2H - {\text{y}}_{b} } \right)\cos \alpha /C_{s} + L\sin \alpha /C_{s} \hfill \\ \Delta t_{9} = \left( {H - {\text{y}}_{b} } \right)/\left( {C_{p} \cos \beta } \right) + \left[ {H - \left( {H - {\text{y}}_{b} } \right)\left. {\tan \alpha \tan \beta } \right]\cos \alpha /C_{s} + L\sin \alpha /C_{s} } \right. \hfill \\ \end{gathered} \right. $$

In summary, by substituting Eqs. (12) through (23) into Eqs. (9) through (11), one can calculate the equivalent load that the nodes on each boundary are subjected to. Subsequently, the automatic generation of the viscoelastic artificial boundaries and the loading of the equivalent node load can be realized by programming APDL language in ANSYS, so as to complete the input of seismic waves on the finite element model.

## Validation of oblique incident seismic wave input method

**Figure 6:**
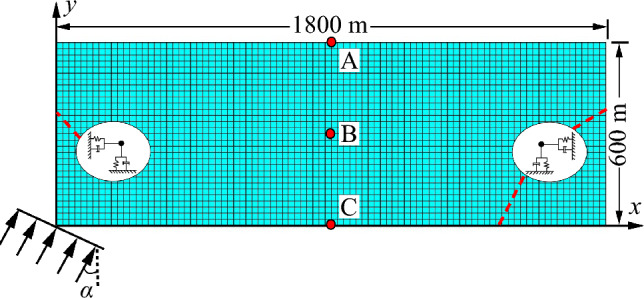
Finite element model of uniform elastic half-space.

To verify the correctness of the input program for SV wave oblique incidence, a finite region measuring 1800 × 600 m was taken from the infinite domain foundation as a uniform elastic half-space model, as depicted in Fig. 6. The upper boundary of the model is a free surface, while viscoelastic artificial boundaries are established on the left, right, and bottom boundaries accordingly. The key parameters of the model are as follows: elastic modulus *E* = 1.25 GPa, poisson ratio *μ* = 0.3, and density *ρ* = 2000 kg/m^3^. Additionally, three observation points, A, B and C, were set at the depth of 0 m, 300 m and 600 m of the model symmetry axis.

**Figure 7:**
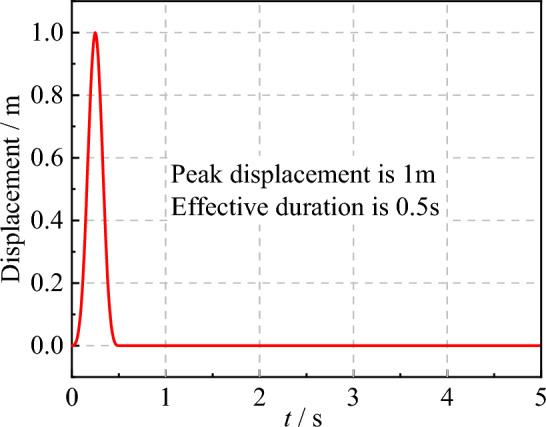
Displacement–time history of incident wave.

The seismic wave velocities were calculated to be $C_{s}$ = 500 m/s and $C_{p}$ = 866 m/s, respectively. The incident SV wave was tested at angles of 0° and 20°. The displacement time history of incident wave is shown in Fig. 7, while the displacement expression of incident wave is shown in Eq. (24):

24 $$ \left\{ \begin{gathered} u(t) = 16\left[ {G(\tau ) - 4G\left( {\tau - 0.25} \right) + 6G\left( {\tau - 0.5} \right) - } \right.\;\left. {4G\left( {\tau - 0.75} \right) + G(\tau - 1)} \right] \hfill \\ G(\tau ) = \tau^{3} H(\tau )\; \hfill \\ \;\;\tau = \frac{t}{T} \hfill \\ \end{gathered} \right. $$

where *T* = 0.5 s is the load duration, *H*(*τ*) is the Heaviside function.

**Figure 8:**
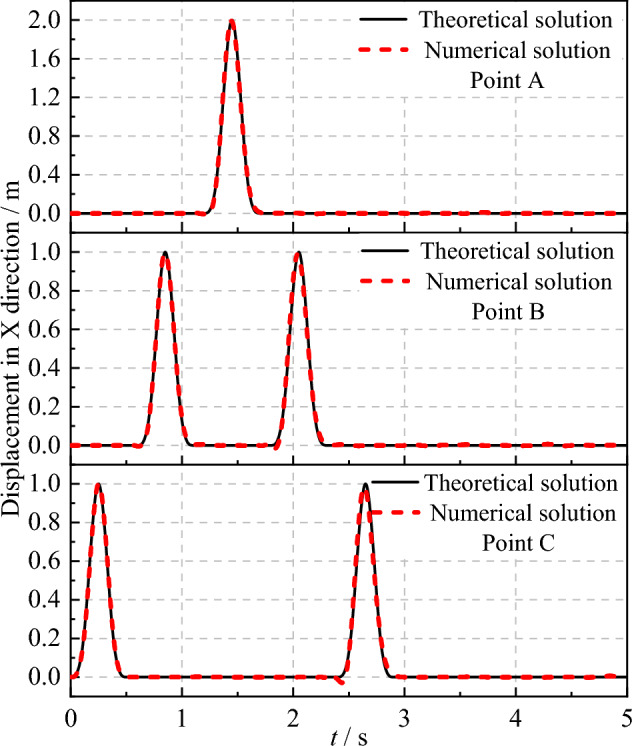
Displacement time history at observation points under SV waves with 0° incident angle.

**Figure 9:**
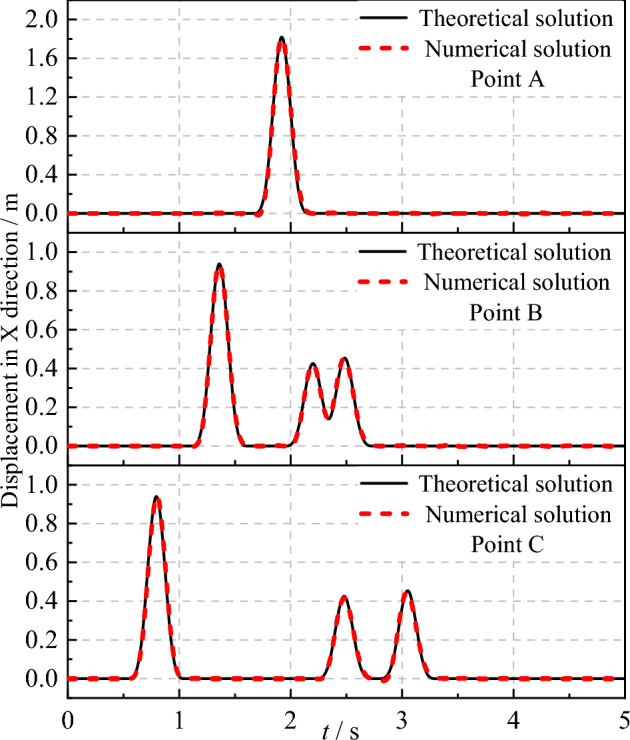
Displacement time history at observation points under SV waves with 20° incident angle.

Figures 8 and 9 present the theoretical and numerical results of the SV wave at observation points A, B, and C in the x-direction when the incident angle is 0° and 20°, respectively. It can be observed from Figs. 8 and 9 that the theoretical and computational displacement time histories at each observation point coincide perfectly, thereby validating the accuracy of the SV wave incidence program developed in this study.

**Figure 10:**
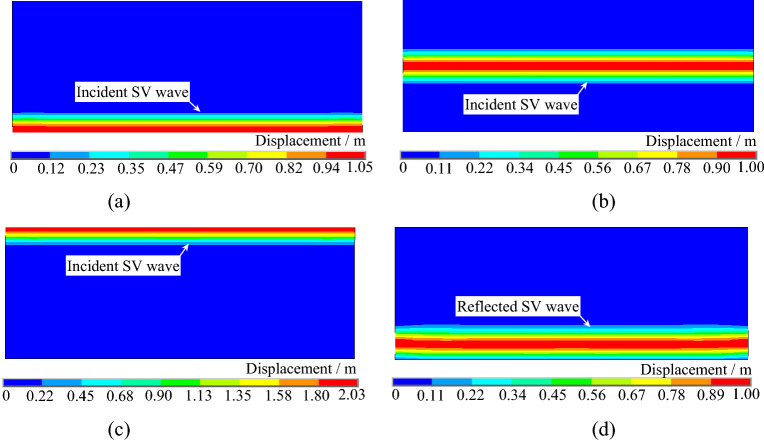
Displacement contour of the uniform semi-infinite space under SV waves with 0° incident angle. (**a**) t = 0.28 s. (**b**) t = 0.85 s. (**c**) t = 1.45 s. (**d**) t = 2.50 s

**Figure 11:**
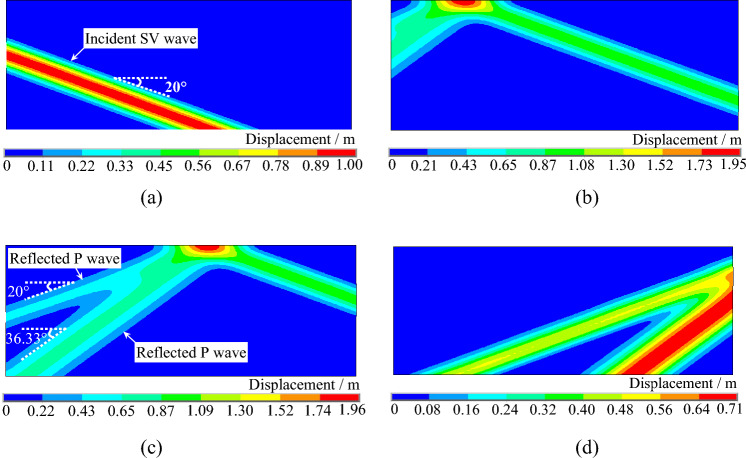
Displacement contour of the uniform semi-infinite space under SV waves with 20° incident angle. (**a**) t = 0.90 s. (**b**) t = 1.60 s. (**c**) t = 2.00 s. (**d**) t = 2.70 s.

Figures 10 and 11 depict the displacement contours of a uniform elastic half space under SV waves incident at 0° and 20° angles, respectively. In Fig. 10, it can be observed that after the SV wave is incident at 0°, a horizontal wavefront propagates upward from the bottom of the model. Upon reaching the top of the model, it reflects at the free surface and propagates downward as a horizontal wavefront until it reaches the bottom of the model again. In Fig. 11, a wavefront tilting at a 20° angle with respect to the horizontal direction propagates upward after the SV wave is incident at 20°. Upon reaching the top of the model, it reflects at the free surface, generating reflected SV waves and reflected P waves. These displacement contours further demonstrate the accuracy of the SV wave oblique incidence program developed in this study.

## Subway station structure calculation model and parameters

This paper uses a two-story three-span subway station structure in a loess site as an example. The station is a reinforced concrete structure with a height of 13.74 m and a width of 21.60 m. The top slab thickness is 0.8 m, the middle slab thickness is 0.4 m, the bottom slab thickness is 0.9 m, and the side wall width is 0.7 m. The middle column has a longitudinal length of 1.2 m, a transverse width of 0.8 m, and a longitudinal spacing of 9.12 m.

**Figure 12:**
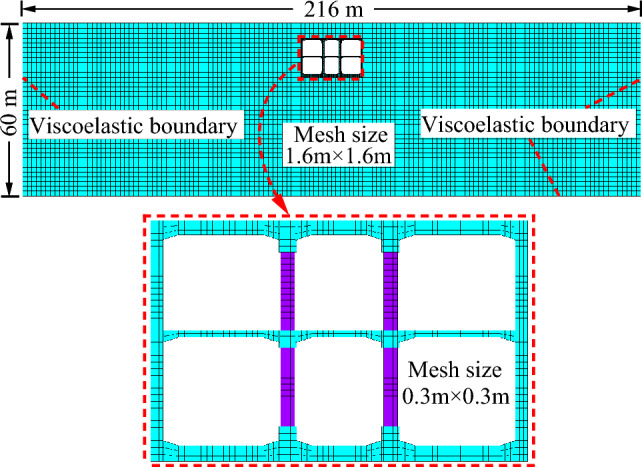
Finite element model of soil-subway station structure interaction.

To eliminate the boundary effect, the width of the model was taken as 216 m (10 times of the structure), the height was 60 m, and the station soil cover thickness was 5 m. The established finite element model of soil-subway station structure interaction is illustrated in Fig. 12. Viscoelastic artificial boundaries were implemented on both sides and the bottom boundary of the model to simulate the radiation damping effect and elastic recovery performance of the infinite domain foundation. The contact behavior between the soil and the subway station structure was simulated using a face-to-face contact approach, with the surface of the station structure designated as the target surface and the surface of the soil designated as the contact surface. Target elements were simulated using TARGE169 elements, and contact elements were simulated using CONTA172 elements. The consideration of opening and sliding between the soil and the station structure was excluded, hence the contact behavior was set to binding. For the simulation, two-dimensional plane strain elements were used to simulate both the soil and the station structure and the ideal elastic–plastic DP material constitutive model (Drucker-Prager), which is used for geotechnical nonlinear analysis in ANSYS software, was adopted to consider the nonlinear characteristics of soil mass and structure^35^. Since the middle columns are not continuously distributed, the equivalent treatment of the middle columns was carried out according to the principle of constant stiffness and mass in the calculation. Accordingly, the elastic modulus and density of the middle columns were reduced according to the method described in literature ^21,37–40^, and the middle columns with equal spacing distribution are equivalent to the shear walls with continuous distribution. Based on the aforementioned studies, it can be inferred that this equivalent method possesses sufficient accuracy to adequately reflect the dynamic response of subway stations. The main material parameters are shown in Table 1, where the parameters for loess are obtained from experiments^35^, and those for walls and plates are selected based on practical engineering experience.

**Table 1:** Main material parameters.

Material	Elastic modulus /GPa	Poisson's ratio	Density /(kg/m^3^)	Cohesion /MPa	Internal friction angle/°
Model soil	0.07	0.3	1650	0.03	22
Wall and sla	31.50	0.2	2500	2.5	55

**Figure 13:**
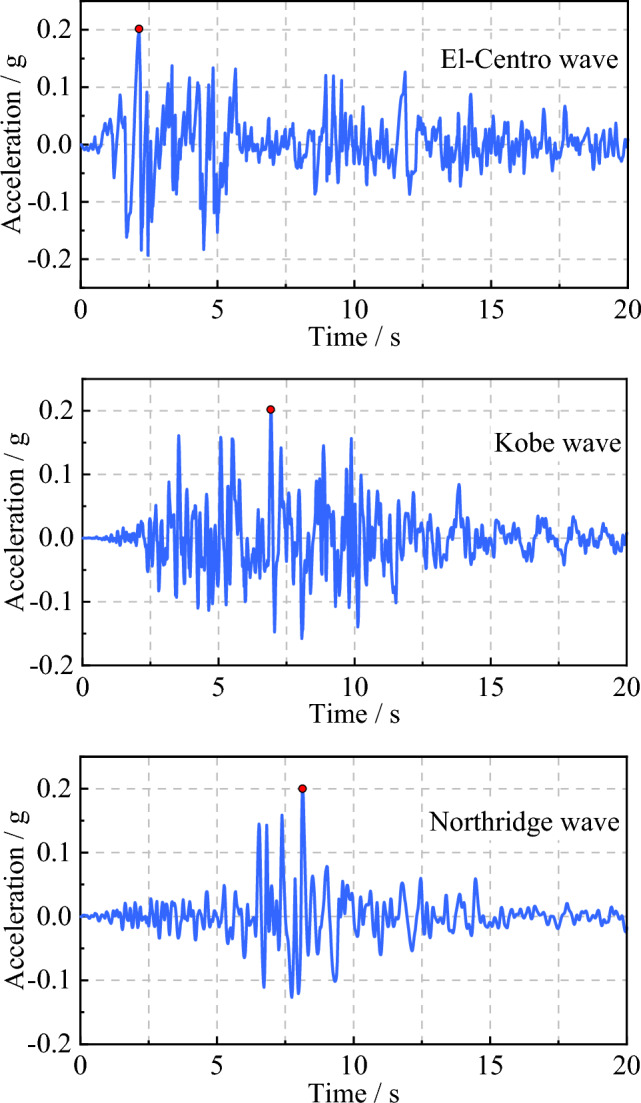
Input seismic acceleration time history.

The El-Centro, Kobe, and Northridge waves were utilized as input ground motions. The duration of each ground motion was 20 s, with a time interval of 0.02 s. Based on the soil parameters, the critical angle of incidence for SV waves is calculated to be 32.31°. Therefore, in the calculations, the angle of incidence *α* for SV waves is set to 0°, 15°, and 30°, respectively. According to the relevant provisions of the Chinese seismic design code regarding the design basic seismic acceleration for building site, the amplitudes of the three ground motion records above were adjusted uniformly to 0.2 g for input^41^. The input seismic acceleration time history is depicted in Fig. 13.

In the calculations, Rayleigh damping was employed to simulate the viscosity of the soil. The relationship between the damping matrix $\left[ {\text{C}} \right]$ and the mass matrix $\left[ {\text{M}} \right]$ and stiffness matrix $\left[ {\text{K}} \right]$ of the system is given by:

25 $$ \left[ C \right] = {\upalpha }_{0} \left[ M \right] + {\upbeta }_{0} \left[ K \right] $$

26 $$ \alpha_{0} = \frac{{2\pi \xi \omega_{i} \omega_{j} }}{{\omega_{i} + \omega_{j} }}\;\;\;\;\;\beta_{0} = \frac{2\xi }{{\omega_{i} + \omega_{j} )}} $$

where *ξ* represents the damping ratio, which is set to 0.05; $\omega_{i}$ and $\omega_{j}$ represent the range of circular frequencies of interest, taken as the first and third natural circular frequencies, respectively.

## Analysis of calculation results

### Displacement response analysis

**Figure 14:**
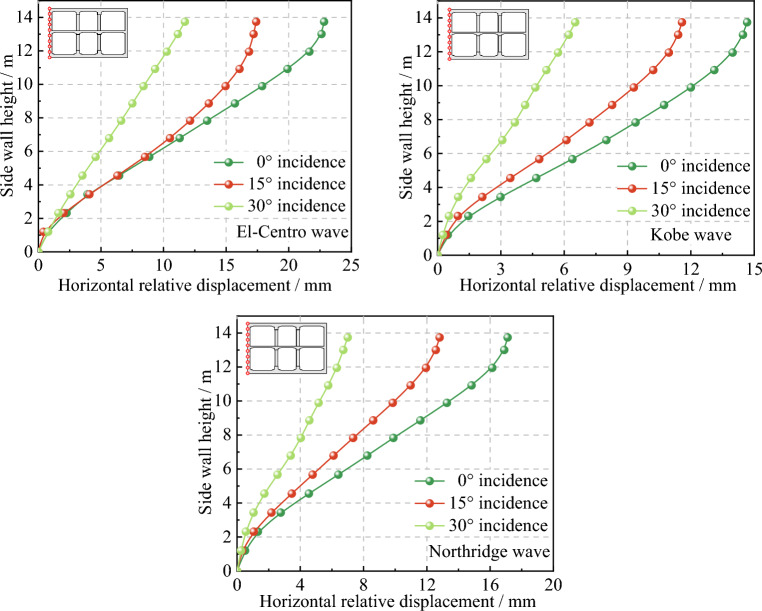
Relative horizontal displacement of subway station side wall.

In order to analyze the variation law of the horizontal relative displacement of the side wall of the subway station structure with the height, some observation points were set along the outer edge of the side wall at various heights. Taking the bottom of the side wall as the base point, the maximum horizontal displacement of each observation point relative to the base point in the process of dynamic time history analysis was extracted, and the results were shown in Fig. 14.

As can be seen from Fig. 14, when inputting different seismic waves, the variation law of horizontal relative displacement of side wall with the height of the structure is basically the same, and all of them show the trend of gradually increasing with the increase of the height of the structure. Considering the incidence angle of seismic waves, there is a gradual decrease in the horizontal relative displacement of the side wall as the seismic wave incidence angle increases. In the lower story of the station structure, the difference in horizontal relative displacement between different incidence angles is minimal. However, as the height of the side wall increases, the discrepancy in horizontal relative displacement between different angles gradually intensifies. This can be attributed to the dynamic amplification effect of the soil surrounding the structure, which increases in tandem with the height.

Table 2 displays the maximum interstory drift ratio $\delta_{\max }$ of the subway station structure under various angles of seismic wave incidence. It is observed that as the incidence angle increases, the maximum interstory drift ratio of the upper, lower, and top-to-bottom stories decreases. Specifically, the interstory drift ratio is most significant at a 0° incidence, with the maximum values being 1/493 for El-Centro wave, 1/801 for Kobe wave, and 1/654 for Northridge wave. All of them fall below the limit value of 1/343 for interstory drift ratio of performance level 2 given in literature^42^. Hence, based on the performance level classification provided in the literature, the subway station structure discussed in this paper can be classified as immediately operational under earthquake conditions with a peak value of 0.2 g. This classification ensures that the critical seismic components of the subway station structure remain predominantly intact, equipment functions are unaffected, and normal operations of the subway station can be maintained. Furthermore, by comparing interstory drift ratios across different stories, it is evident that the lower story exhibits the largest ratio, followed by the top to bottom story, and finally, the upper story at incidences of 0° and 15°. However, when the seismic wave is incident at 30°, the lower story has the smallest interstory drift ratio, while the interstory drift ratios of the upper story and top to bottom story are larger. This indicates that the deformation of the subway station structure is inconsistent under different angles of seismic wave incidence.

**Table 2:** Maximum interstory drift ratio of subway station.

Incident angle	Upper story	Lower story	Top-to-bottom
El-Centro 0°	1/592	1/512	1/516
El-Centro 15°	1/714	1/669	1/686
El-Centro 30°	1/1317	1/1370	1/1281
Kobe 0°	1/884	1/811	1/818
Kobe 15°	1/1107	1/1030	1/1044
Kobe 30°	1/2088	1/2330	1/2256
Northridge 0°	1/762	1/679	1/687
Northridge 15°	1/1014	1/935	1/949
Northridge 30°	1/1915	1/2088	1/1969

**Figure 15:**
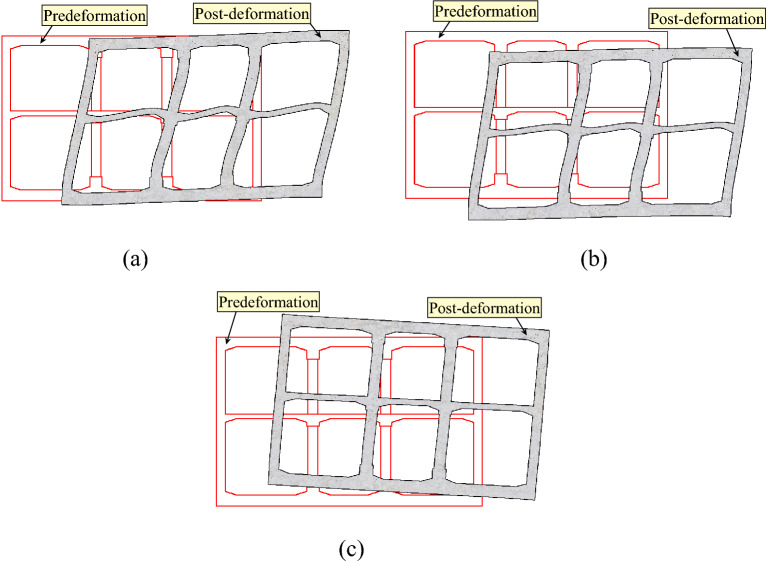
Deformation diagram of the subway station structure. (**a**) α = 0°. (**b**) α = 15°. (**c**) α = 30°.

Figure 15 displays the deformation diagram of the subway station structure at the moment when the maximum horizontal relative displacement occurs under various incidence angles of seismic waves. Only the results for the inputting El-Centro wave are provided here, as the results for the Northridge and Kobe waves are similar. Therefore, repeating them is unnecessary (same below).

As depicted in Fig. 15, the deformation and displacement of the subway station structure differ based on the incidence angles of seismic waves. Specifically, when the seismic wave is vertically incident, when the seismic wave is vertically incident, the horizontal displacement of the subway station structure is greater while the vertical displacement is negligible. Moreover, the station structure experiences transverse shear deformation predominantly. When the seismic wave is incident at a 15° angle, the subway station structure experiences greater displacement in both horizontal and vertical directions, with the station structure mainly undergoing transverse shear deformation. As for an inclination angle of 30°, there is a reduction in horizontal displacement while vertical displacement increases, with vertical shear deformation being the primary cause of the station structure's deformation. Comparing it to vertical incidence, oblique incidence of seismic waves results in noticeable vertical displacement of the subway station structure. Additionally, the subway station structure exhibits non-uniform deformation under various incidence angles. Smaller incidence angles primarily induce transverse shear deformation, while larger incidence angles predominantly provoke vertical shear deformation in the station structure.

### Acceleration response analysis

**Figure 16:**
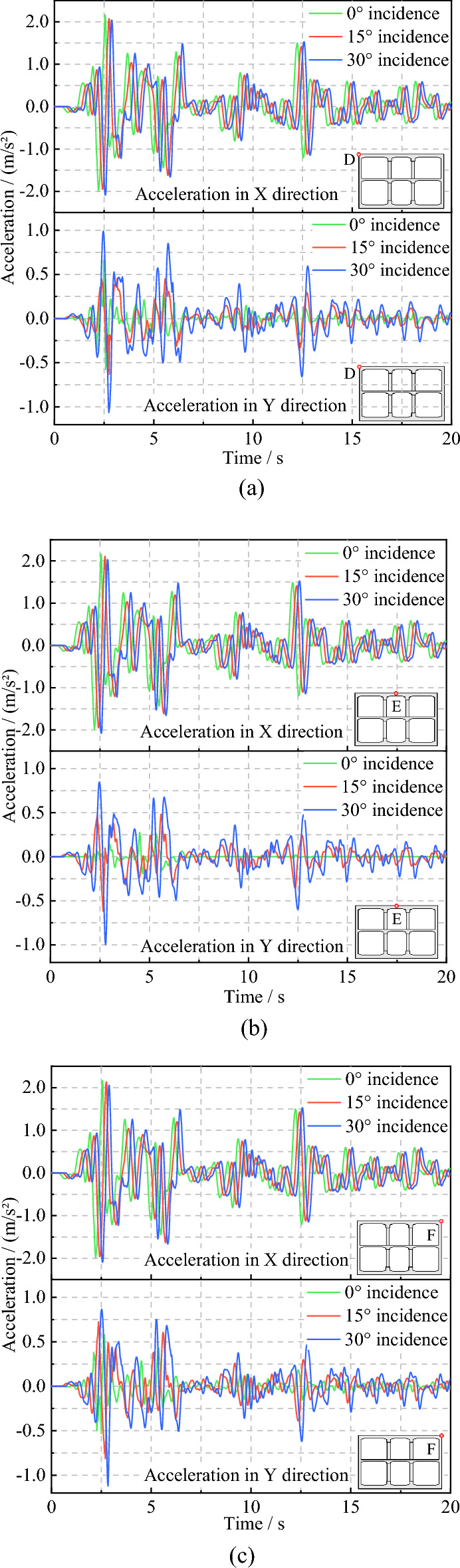
Acceleration time history of D, E, F points. (**a**) Observation points D. (**b**) Observation points E. (**c**) Observation points F.

Three observation points, labeled D, E, and F, were positioned at the left, center, and right locations of the top slab in the subway station structure. The horizontal and vertical acceleration time histories (in the x and y-directions, respectively) of these three points were extracted when the seismic wave was incident at different angles. The outcomes are presented in Fig. 16.

As shown in Fig. 16, when examining the horizontal acceleration time history, the peak acceleration values of observation points D, E, and F fluctuate slightly as the incidence angle of seismic waves increases. However, the overall change is not significant and can be disregarded. Additionally, the acceleration curves corresponding to different incidence angles show a similar shape. From the perspective of the vertical acceleration time history, it is evident that with an increase in the incidence angle of the seismic wave, the peak acceleration of the three observation points, D, E and F, increases significantly. It can be seen that, compared to vertical incidence, oblique incidence of seismic waves results in a significant increase in the vertical acceleration of the structure. Furthermore, as the oblique incidence angle becomes more significant, this effect is more apparent. Conversely, the change in the incidence angle of the seismic waves has a negligible effect on the horizontal acceleration of the structure.

**Figure 17:**
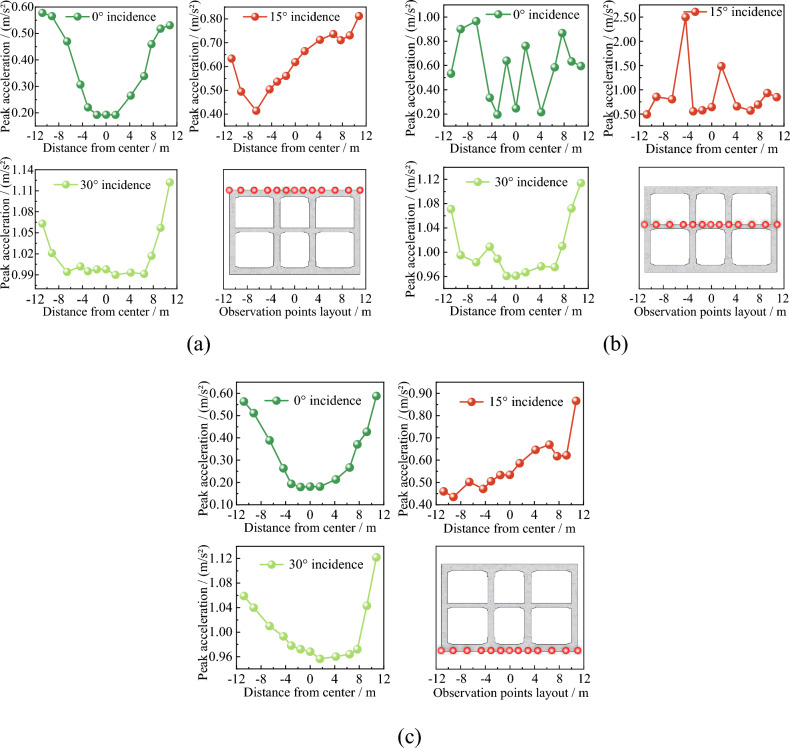
Distribution of peak acceleration at subway station. (**a**) Peak acceleration of the top slab. (**b**) Peak acceleration of the middle slab. (**c**) Peak acceleration of the bottom slab.

In order to analyze the distribution characteristics of peak acceleration in subway station structures, the absolute values of peak vertical acceleration at specific nodal points on the top, middle, and bottom slabs were extracted. The obtained results are presented in Fig. 17.

From Fig. 17, it is evident that when the seismic wave is vertically incident, the peak acceleration on the top slab, middle slab, and bottom slab exhibits symmetric distribution along the structure's centerline. Both the top and bottom slabs demonstrate a "U"-shaped symmetrical distribution, with greater peak acceleration further away from the structure's centerline. For the middle slab, the peak acceleration exhibits an irregular symmetrical distribution shape. Specifically, the peak acceleration is relatively small at the junction between the middle slab and the middle column, as well as at the center of the middle slab, while it is larger at the center of the left and right span. When the seismic wave is inclined at a 15° angle, the peak acceleration on the roof, middle slab, and bottom slab demonstrates the most asymmetrical distribution. Specifically, the peak acceleration on the right side of the structure is greater than that on the left side for both the top and bottom slabs. As for the middle slab, the maximum peak acceleration takes place at the intersection of the right span and the middle column. When the seismic wave is inclined at a 30° angle, the peak acceleration on the top slab, middle slab, and bottom slab gradually approaches symmetrical distribution, with the maximum peak acceleration appearing on the right wall of the structure. It is worth noting that, except for some nodes, the peak acceleration on the top slab, middle slab, and bottom slab increases with greater incidence angle of the seismic wave. In summary, compared to vertical incidence, inclined incidence of seismic waves induces non-uniform loads on the subway station structure. This, in turn, leads to asymmetric acceleration responses in an otherwise symmetrical structure, resulting in significant variations in stress and deformation across different parts of the station structure.

### Stress response analysis

**Figure 18:**
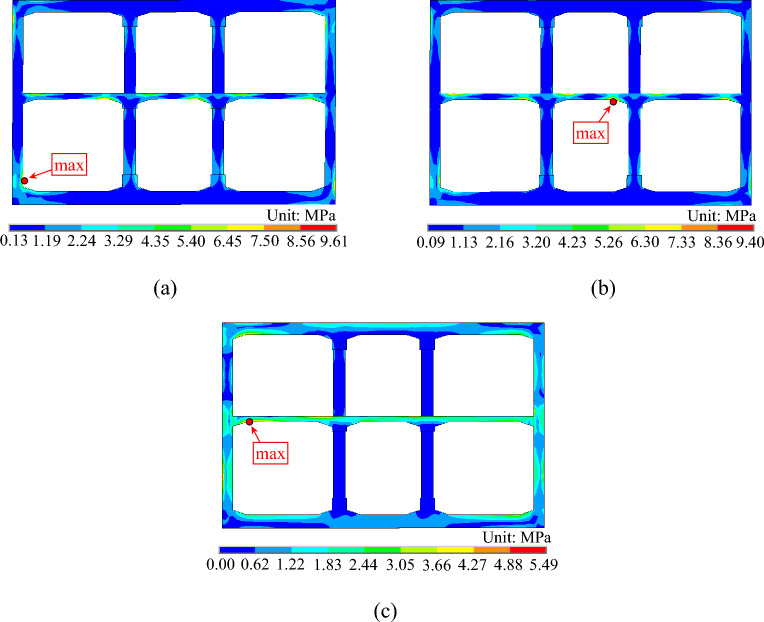
Mises stress nephogram of subway station. (**a**) α = 0°. (**b**) α = 15°. (**c**) α = 30°.

After comparing the calculated results, it was discovered that the stress response in the subway station structure does not reach its peak value at the moment when the maximum horizontal relative displacement occurs between the top and bottom slab of the structure. Thus, the stress distribution during this moment cannot fully represent the peak stress state of the subway station structure. The stress response analysis must extract peak stress results during dynamic time history analysis. Figure 18 displays the stress nephogram when Mises stress reaches its maximum value at the subway station structure with different incidence angles of seismic waves.

From Fig. 18, it is evident that for the seismic wave incident at 0° (vertical incidence) and 15° angles, the Mises stresses are greater at the junctions of the slab and side wall, slab and middle column, and at the two ends of the middle column. In contrast, Mises stresses are lower in the remaining parts of the structure. The highest stresses occur at the junction of the side wall and bottom slab and at the junction of the middle slab and middle column respectively. When the seismic wave is incident at a 30° angle, the Mises stresses exhibit greater magnitude at the junction of the top and bottom slabs with the side wall, the middle of the side wall, and throughout the whole range of the middle slab. The peak stress is reached at the junction of the middle slab and the side wall. According to the analysis, weak parts under seismic action include the junction between the slab and the side wall, the slab and middle column, and the ends of the middle column. Therefore, special attention should be paid to these areas during seismic design. Furthermore, the subway station structure experiences a decrease in peak Mises stress as the incident angle of seismic waves increases, and the stress response is greatest when the seismic wave is incident vertically.

### Structural damage analysis

**Figure 19:**
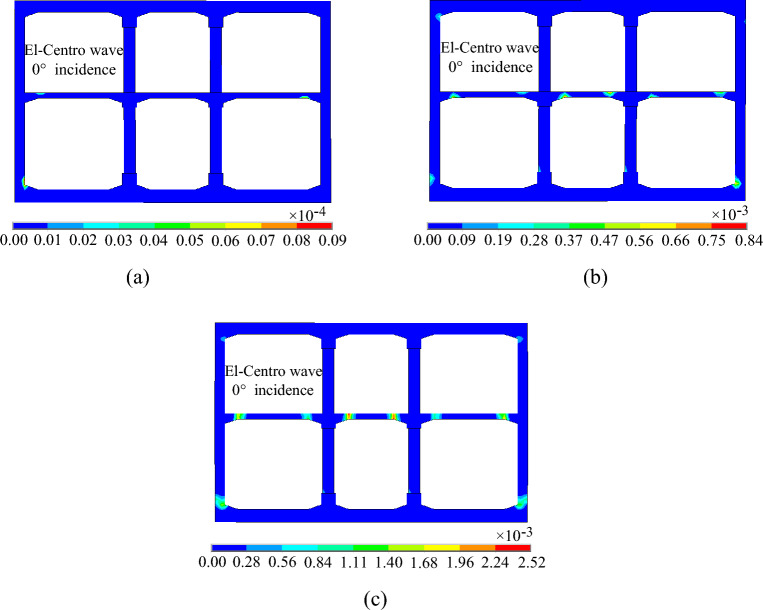
Equivalent plastic strain of the subway station under SV waves with 0° incident angle. (**a**) t = 1.50 s. (**b**) t = 2.20 s. (**c**) t = 20.00 s.

**Figure 20:**
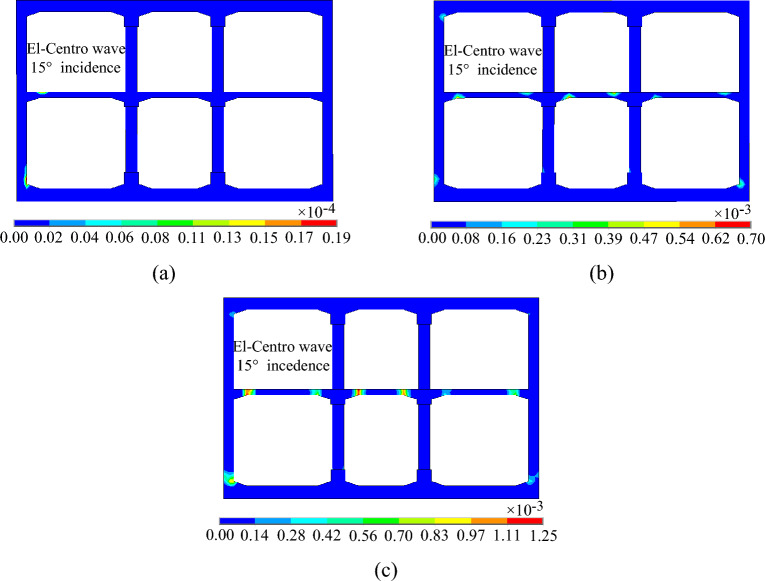
Equivalent plastic strain of the subway station under SV waves with 15° incident angle. (**a**) t = 2.14 s. (**b**) t = 2.60 s. (**c**) t = 20.00 s.

**Figure 21:**
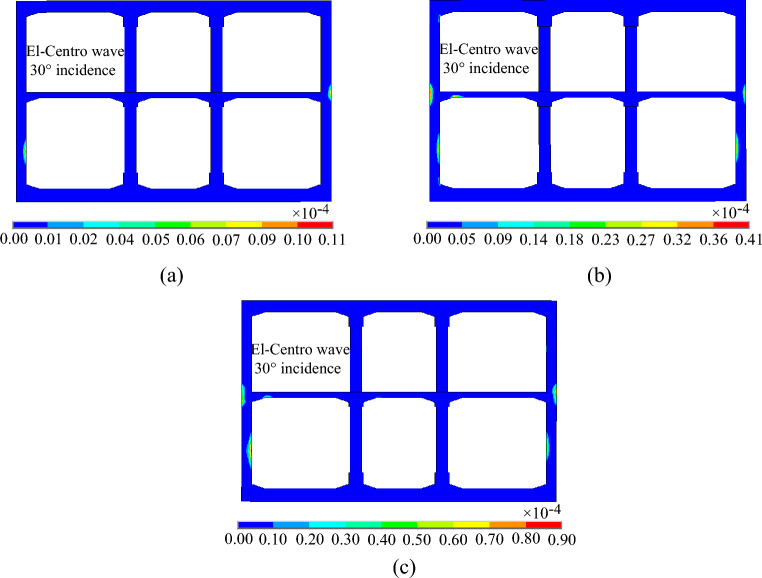
Equivalent plastic strain of the subway station under SV waves with 30° incident angle. (**a**) t = 2.46 s. (**b**) t = 5.00 s. (**c**) t = 20.00 s.

Figures 19, 20 and 21 shows the evolution process of the equivalent plastic strain of the subway station structure under varying incidence angles of seismic waves. It can be observed that the evolution trend of equivalent plastic strain of the subway station structure is basically the same when the seismic wave is incident at 0° and 15°. Initially, plastic deformation transpires at the junctions of the middle slab and the side wall, middle slab and middle column, and at the bottom of the side wall. The plastic deformation is localized and appears only on the structure surface. As the ground motion continues, the plastic deformation at the junctions of the middle slab and the side wall, the middle slab and the middle column gradually develops to the section center of the middle slab, the plastic deformation at the bottom of the side wall gradually develops to the section center of the side wall. Simultaneously, the plastic deformation begins to appear at the junction of the side wall and the top slab. After the seismic wave action for 20 s, the plastic deformation of the middle slab and the side wall basically runs through the whole section, and the small plastic deformation appears at the bottom of the lower story middle column. In the case of a 30° incident angle of the seismic wave, the plastic deformation first appears at the middle of the lower story side wall and the junction of the middle slab and the side wall, and then the plastic deformation gradually develops towards the center of the section, but does not run through the whole section. From the incident angle of seismic wave, the equivalent plastic strain of subway station structure decreases gradually with the increase of seismic wave incidence angle, which indicates that the plastic deformation of subway station structure is the largest when the seismic wave is vertically incident.

## Conclusions

The paper discusses the seismic response characteristics of a two-story three-span subway station structure under different angle incidences of SV waves, using numerical simulation. It was discovered that the seismic wave incidence angle greatly affects the subway station structure's seismic response. The following are the primary findings:

1. Compared to vertical incidence, oblique incidence of seismic waves causes noticeable displacement in the vertical direction of subway station structures. Furthermore, the structure produces non-uniform deformation at varying angles of incidence. When the incidence angles of the seismic waves are small, the station structure mainly undergoes transverse shear deformation, while for large incidence angles, the vertical shear deformation is predominant.
2. Oblique incidence of seismic waves, compared to vertical incidence, significantly increases the vertical acceleration of the subway station structure. Moreover, the greater the angle of the oblique incidence, the more significant the increase effect is. However, the change in the angle of seismic wave incidence has an insignificant impact on the horizontal acceleration of the structure.
3. Inclined incidence of seismic waves creates non-uniform loads on the subway station structure, leading to asymmetric acceleration responses in the symmetrical structure. Consequently, significant differences arise in the stress and deformation of various parts of the station structure.
4. The stress response of a subway station structure does not reach its peak at the moment when the maximum horizontal relative displacement occurs between the top and bottom of the structure. Moreover, the stress distribution during this moment cannot entirely represent the peak stress state of the subway station structure.
5. Compared with other parts of subway station structure, the junctions of the slab and the side wall, the slab and the middle column, as well as the two ends of the middle column, have greater stress response and plastic deformation, which are the weak parts under earthquake action, and should be paid more attention to in seismic design.

## Data Availability

Except for the source code of the program written in this paper due information involving trade secrets, personal privacy, and other disclosure that may harm the legitimate rights and interests of third parties shall not be disclosed, all other experimental data are available upon request from the corresponding author.
